# Machine learning application to predict in-situ stresses from logging data

**DOI:** 10.1038/s41598-021-02959-9

**Published:** 2021-12-06

**Authors:** Ahmed Farid Ibrahim, Ahmed Gowida, Abdulwahab Ali, Salaheldin Elkatatny

**Affiliations:** 1 College of Petroleum Engineering and Geosciences, King Fahd University of Petroleum and Minerals, Dhahran, 31261 Saudi Arabia; 2 College of Petroleum Engineering and Geosciences, King Fahd University of Petroleum and Minerals, Dhahran, 31261 Saudi Arabia

**Keywords:** Energy science and technology, Engineering, Mathematics and computing

## Abstract

Determination of in-situ stresses is essential for subsurface planning and modeling, such as horizontal well planning and hydraulic fracture design. In-situ stresses consist of overburden stress (σ_v_), minimum (σ_h_), and maximum (σ_H_) horizontal stresses. The σ_h_ and σ_H_ are difficult to determine, whereas the overburden stress can be determined directly from the density logs. The σ_h_ and σ_H_ can be estimated either from borehole injection tests or theoretical finite elements methods. However, these methods are complex, expensive, or need unavailable tectonic stress data. This study aims to apply different machine learning (ML) techniques, specifically, random forest (RF), functional network (FN), and adaptive neuro-fuzzy inference system (ANFIS), to predict the σ_h_ and σ_H_ using well-log data. The logging data includes gamma-ray (GR) log, formation bulk density (RHOB) log, compressional (DTC), and shear (DTS) wave transit-time log. A dataset of 2307 points from two wells (Well-1 and Well-2) was used to build the different ML models. The Well-1 data was used in training and testing the models, and the Well-2 data was used to validate the developed models. The obtained results show the capability of the three ML models to predict accurately the σh and σH using the well-log data. Comparing the results of RF, ANFIS, and FN models for minimum horizontal stress prediction showed that ANFIS outperforms the other two models with a correlation coefficient (R) for the validation dataset of 0.96 compared to 0.91 and 0.88 for RF, and FN, respectively. The three models showed similar results for predicting maximum horizontal stress with R values higher than 0.98 and an average absolute percentage error (AAPE) less than 0.3%. a^20^ index for the actual versus the predicted data showed that the three ML techniques were able to predict the horizontal stresses with a deviation less than 20% from the actual data. For the validation dataset, the RF, ANFIS, and FN models were able to capture all changes in the σ_h_ and σ_H_ trends with depth and accurately predict the σ_h_ and σ_H_ values. The outcomes of this study confirm the robust capability of ML to predict σ_h_ and σ_H_ from readily available logging data with no need for additional costs or site investigation.

## Introduction

In-situ stresses are presented in overburden stress (${\upsigma }_{\mathrm{V}}$), and horizontal stresses, named minimum (${\upsigma }_{\mathrm{h}})$ and maximum (${\upsigma }_{\mathrm{H}}$) horizontal stresses. The stress state of the earth's subterranean formations is considered one of the essential information that has a great interest in petroleum engineering and geoscience fields. Therefore, fully understanding the stress state is crucial for well orientation and hydraulic fracture design that may significantly affect oil and gas production. For example, a horizontal gas well drilled in the direction of the ${\upsigma }_{\mathrm{h}}$ direction of Marcellus shale has a 40–50% increase in productivity compared to a 45° off-azimuth well. Moreover, ${\upsigma }_{\mathrm{h}}$ is essential for geomechanical evaluation, such as wellbore stability^1^. Furthermore, different seismic studies in the Permian basin showed the importance of ${\upsigma }_{\mathrm{h}}$ in controlling hydraulic fracture propagation. Hence, horizontal stresses should be estimated accurately to optimize drilling and completion operations and improve the hydraulic fracturing process. The challenging task in the in-situ stress analyses is the estimation of the ${\upsigma }_{\mathrm{h}}\mathrm{ and }{\upsigma }_{\mathrm{H}}$, because the ${\upsigma }_{\mathrm{V}}$ can be directly computed by integrating the rock density with depth using the density logs^2^.

The stress distribution around the borehole directly impacts the drilling operation and the associated wellbore instability, affecting the wellbore integrity and causes many drilling-related issues such as lost circulation, pack-off, stuck bottom-hole-assembly (BHA) or casing^3,4^. Thus, a comprehensive geomechanical model of downhole formations helps address many issues through different stages of reservoir life. An example of these issues are maintaining borehole stability in drilling operations, formation instability within the production operations and sand production, and the applicable wellbore completion design selection^5–10^.

The ${\upsigma }_{\mathrm{h}}$ can be estimated through different injection tests. These tests include Minifrac, Leak-off Test (LOT), eXtended Leak-off Test (XLOT), or Diagnostic Fracture Injection Test (DFIT)^10–13^. The ${\upsigma }_{\mathrm{H}}$ cannot be directly measured using these methods^14^, and theoretical and empirical correlations are required to estimate the ${\upsigma }_{\mathrm{H}}$ depending on the values of ${\upsigma }_{\mathrm{V}}$ and ${\upsigma }_{\mathrm{h}}$^15,16^. The main challenges for the direct measurements are time-consuming and high cost. Besides, it would not provide a continuous profile of the least principal stresses because such tests are typically performed at specific depths.

Numerical modeling depends on the finite different and finite element systems that can be used to model the in-situ stresses. Other physics-based theoretical models, such as uniaxial strain theory, poroelastic strain models, were developed to determine the in-situ stresses of the downhole formations^10,17–19^. These models depend on having measurements of some in-situ geomechanical parameters, static elastic modulus, static Poisson's ratio, and elastic strains. The most accurate method for obtaining such measurements is retrieving core samples from the downhole formations and conducting triaxial tests to estimate these parameters^19^. The measured parameters would be correlated to the conventional logging data to develop a continuous profile before applying the aforementioned models. Besides, at least one direct field test, i.e., leak-off test, should be conducted to calibrate these profiles and include the effect of the tectonic stresses so that they would effectively represent the in-situ stress state of the downhole formations^20–22^. However, the main limitation of this technique is that retrieving such core samples is an expensive and laborious process that makes such measurements not usually available for all drilled wells.

## Applications of machine learning in geomechanics

Machine learning (ML) models can be built to predict specific parameters as a function of available logging data without adding more cost or well intervention. Different ML techniques such as RF, ANFIS, FN, ANN, and support vector machine (SVM), have been applied in the petroleum industry^23–26^. Acar and Kaya applied SVM to estimate the elastic modulus for weak rocks. They found that the SVM model was more effective than the multiple regression process^23^. Similarly, Gowida et al. used different ML techniques including ANN, ANFIS, and SVM to calculate the unconfined compressive strength (UCS) from the drilling data. They found that using ML techniques to predict UCS was superior compared to the available empirical correlations^24^. Moreover, ML were used to calculate the oil production rate as a function of the choke parameters^26^. ANFIS and FN models outperform the available empirical correlation to forecast oil production, especially in complex systems with high gas-oil ratios and high water cut.

Bayesian Network (BN) and random forest (RF) models were used to estimate the ground vibration result from the quarry blasting^27^. RF model showed higher accuracy results compared to the BN model, where the accuracy level was 90% for the RF model comparing to 87% for the BN model for the testing dataset. Zhou et al. used different ML to forecast the shear strength of rockfill materials^28^. The results revealed that RF models were capable to produce accurate results for the shear strength comparing to the ANN model and multiple regression analysis, and the R-value was 0.98 from the RF model comparing to 0.96 for the ANN model. Similarly, ANFIS and ANN models predicted UCS and Young’s modulus^29^. ANFIS model showed a superior behavior comparing to the ANN model and multiple regression process. R^2^ for the ANFIS model was 0.99 comparing to 0.91, and 0.6 for the ANN model and the multiple regression analysis, respectively^29^. Another ANFIS model was developed to calculate the bearing capacity of the cohesive soft soils^30^. The ANFIS model revealed an R value of 0.99, and 0.96 between the actual and the estimated data for the training, and testing datasets, respectively. Based on this analysis, random forest and ANFIS machine learning revealed a superior performance to predict the geomechanical parameters.

In-situ stress determination is crucial for well planning and hydraulic fracture designing. Borehole injection tests or theoretical methods can be used to estimate the in-situ stresses. However, these methods are complex, expensive, need unavailable tectonic stress data, and only can be conducted at a specific depth. Therefore, this study aims to apply different machine learning tools to predict the ${\upsigma }_{\mathrm{h}}$ and ${\upsigma }_{\mathrm{H}}$ and provide a continuous profile of in-situ stress using the conventional well-log data with no additional cost or well intervention. The logging data consist of gamma-ray (GR) log, formation bulk density (RHOB) log, compressional (DTC), and shear (DTS) wave transit-time log. Two wells with 2307 data were used to build and validate the RF, ANFIS, and FN models.

## Methodology

### Data description

Well-logs data from two wells were collected from the Middle East region representing a complex carbonate formation. A dataset of 2307 points included GR, DTC, DTS, RHOB, neutron porosity (NPHI) log, dynamic elastic modulus (Ed), dynamic Poisson's ratio (PRd), and the corresponding ${\upsigma }_{\mathrm{h}}$ and ${\upsigma }_{\mathrm{H}}$. Table 1 summarizes the statistical analysis of the dataset from both wells, including mean, symmetry coefficients (skewness), and the quartiles of each parameter. Most of the parameters have a positive skewness factor, except ${\upsigma }_{\mathrm{H}}$ has a negative skewness factor, with most of the data concentrated to the upper end of the ${\upsigma }_{\mathrm{H}}$ range. The ${\upsigma }_{\mathrm{h}}$ and ${\upsigma }_{\mathrm{H}}$ gradients are recorded in psi/ft to account for the change of stresses with depth.

**Table 1:** Statistical analysis for the collected dataset for the two wells.

	GR (API)	DTC (µs/ft)	DTS (µs/ft)	RHOB (g/cm^3^)	NPHI	PRd	Ed (psi)	${\upsigma }_{\mathrm{h}}$(psi)	${\upsigma }_{\mathrm{H}}$(psi)	${\upsigma }_{\mathrm{h}}$(psi/ft)	${\upsigma }_{\mathrm{H}}$(psi/ft)
Mean	29	49	90	2.8	0.30	0.30	84,534	11,851	13,724	0.88	1.02
Standard deviation	14	3	7	0.1	0.01	0.01	11,203	300	485	0.00	0.02
Minimum	3	45	81	2.3	0.28	0.28	33,367	11,240	12,308	0.87	0.94
Quartile 25%	17	47	86	2.8	0.29	0.29	77,235	11,616	13,395	0.88	1.01
Quartile 50%	28	47	88	2.8	0.29	0.29	87,839	11,864	13,803	0.88	1.02
Quartile 75%	37	50	94	2.9	0.30	0.30	92,950	12,082	14,044	0.88	1.03
Maximum	90	66	132	3.0	0.32	0.33	101,968	12,361	14,599	0.89	1.05
Skewness	0.68	2.43	2.43	− 0.87	0.95	1.36	− 1.37	− 0.14	− 0.41	0.11	− 1.09

Since large numbers of input features may cause poor performance for ML models, the redundant and irrelevant features were removed, considering not incurring much loss of information. A dimensionality reduction process was applied to simplify the developed models. The correlation coefficient (R value) was calculated between the inputs to evaluate the dependency of each input on each other, and the following equation was used to calculate the R value.

1 $$\mathrm{R}=\frac{\left[\frac{\sum \left({\mathrm{x}}_{\mathrm{i}}-{\upmu }_{\mathrm{x}}\right)\left({\mathrm{y}}_{\mathrm{i}}-{\upmu }_{\mathrm{y}}\right)}{\mathrm{N}-1}\right]}{{\upsigma }_{\mathrm{x}}{\upsigma }_{\mathrm{y}}}$$

where R is the Pearson’s correlation coefficient between the dependent parameters ${\mathrm{y}}_{\mathrm{i}}$ and independent parameters, ${\mathrm{x}}_{\mathrm{i}}$. The ${\upmu }_{\mathrm{x}}\mathrm{ and }{\upmu }_{\mathrm{y}}$ are the mean values of the independent and dependent parameters, respectively. The ${\upsigma }_{\mathrm{x}}\mathrm{ and }{\upsigma }_{\mathrm{y}}$ are the standard deviation of the independent and dependent parameters, respectively.

**Figure 1:**
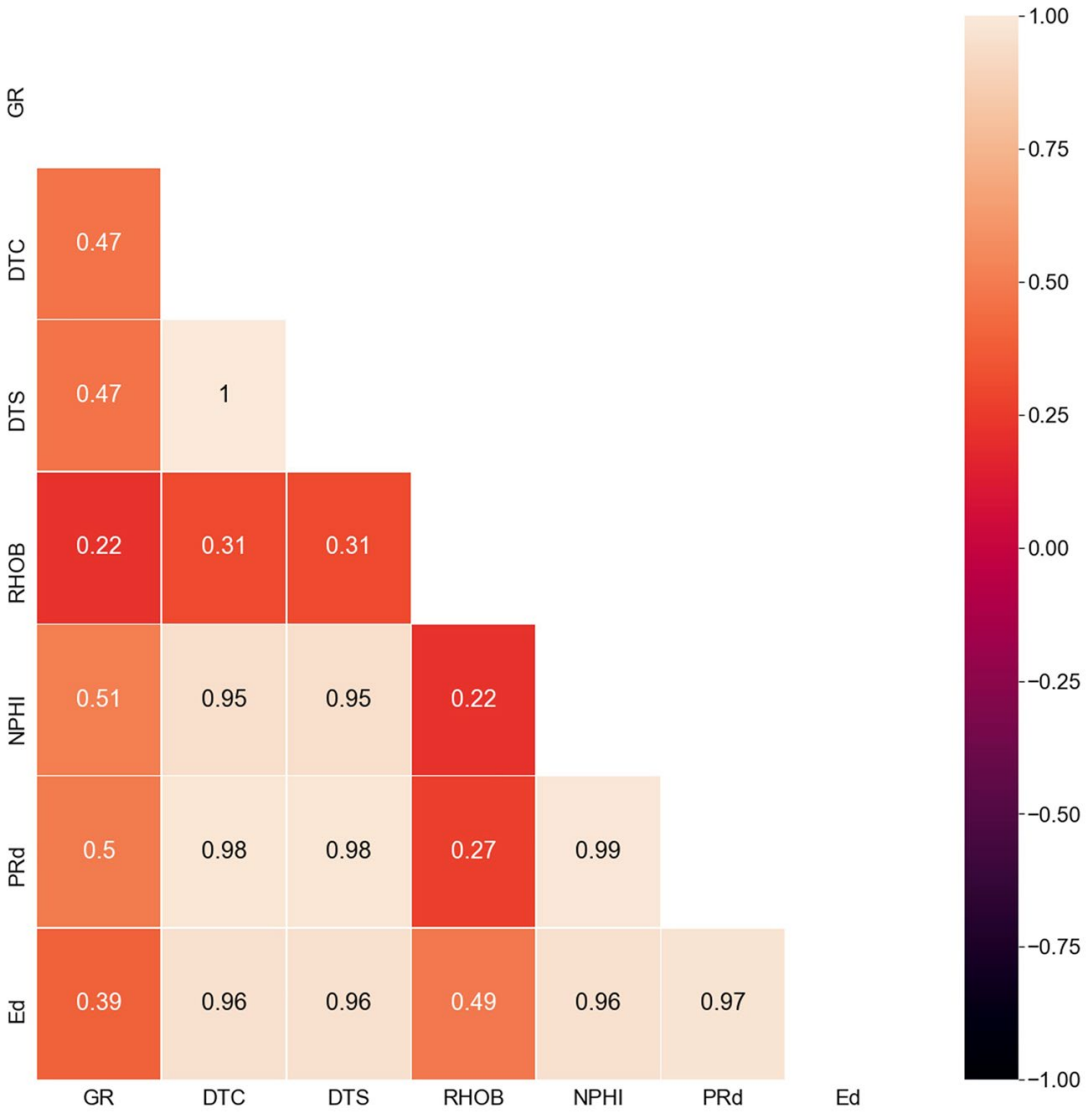
A heat map for the absolute value for the correlation coefficient between the input parameters with each other.

Figure 1 shows a heat map for the absolute value for the correlation coefficient between the input parameters with each other. A high absolute R-value between two parameters indicates a high dependency on each other, hence, only one of them would be considered, and the others would be excluded. Therefore, only GR, RHOB, DTS, and DTC were selected to feed the proposed models, while the other input features were excluded since they depend on the selected ones. Moreover, Sensitivity analysis for the input parameters in the model performance was conducted to judge the model performance with the selected input features as will be discussed in detail in “Result”*** section.

**Figure 2:**
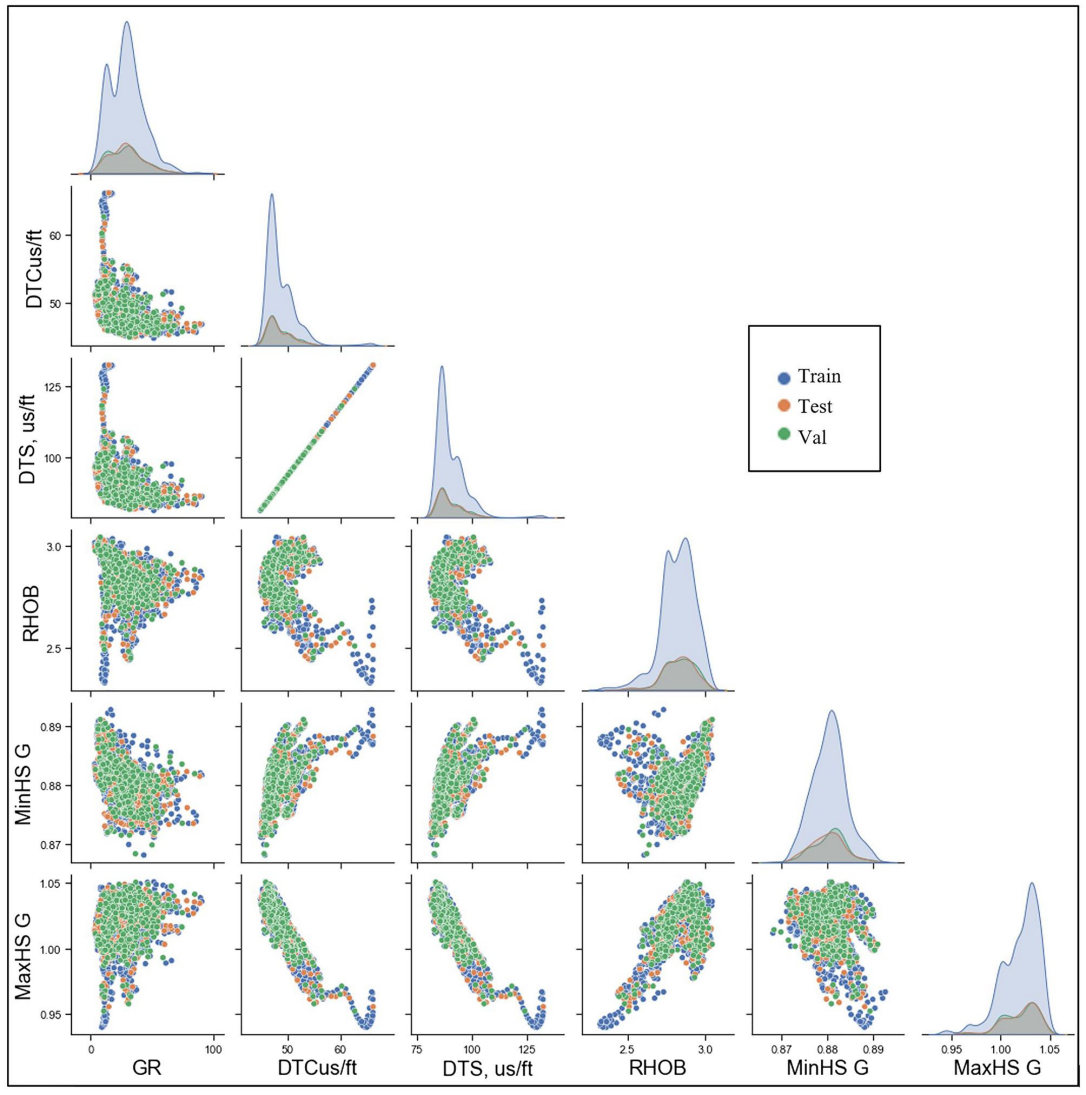
Scatter matrix plot of data features. Blue dots for the training dataset, orange dots for the testing dataset, and green dots for the validation dataset. The diagonal charts show the distribution of each parameter.

Figure 2 shows a scatter matrix plot for the data features and how two properties vary and give insights into whether there is a direct correlation between the properties. The diagonal charts show the distribution of each parameter. Most of the parameters showed a slightly positive skewness, where the mass of the distribution is concentrated on the left (lower values). The ${\upsigma }_{\mathrm{h}}$ gradient is normally distributed, whereas the ${\upsigma }_{\mathrm{H}}$ gradient showed negative skewness with a long tail due to the left side of the graph. Figure 2 shows that the validation set, in green, is located within the training and testing sets in the different parameters.

**Figure 3:**
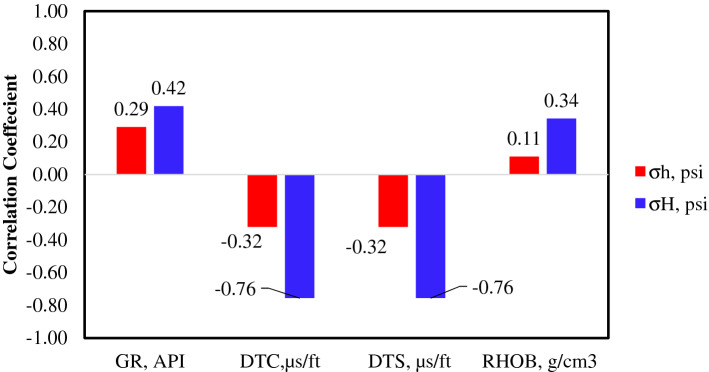
Correlation coefficient between the conventional logging parameters and the minimum and maximum (${\sigma }_{h}$ and ${\sigma }_{H}$) horizontal stresses.

Pearson's correlation coefficient was used to explore the importance of each of the input parameters in the prediction of minimum and maximum horizontal stress. Equation 1 was used to calculate the R values, where ${\mathrm{y}}_{\mathrm{i}}$ was the ${\upsigma }_{\mathrm{h}}$ and ${\upsigma }_{\mathrm{H}}$, and ${\mathrm{x}}_{\mathrm{i}}$ is the input paramters (GR, RHOB, DTS, and DTC). Figure 3 present the correlation coefficient for ${\upsigma }_{\mathrm{h}}$ and ${\upsigma }_{\mathrm{H}}$ with the input parameters. GR and RHOB showed a positive relationship with the stresses (psi). RHOB reflects the overburden stress, which is directly related to the minimum and maximum horizontal stress. GR reflects the clay and shale concentrations that increase the formation stresses. DTS and DTC reflect the formation compaction. With high formation stresses, the formation grains' compaction increases. Hence, the DTC, and DTS decreases, which was indicated by a negative correlation coefficient between the DTC and DTS, and ${\upsigma }_{\mathrm{h}}$ and ${\upsigma }_{\mathrm{H}}$.

### Models development

The dataset from Well-1 (1614 points) was used to develop models with training to testing data ratio of 75/25. The unseen dataset from Well-2 (693 points) was used to validate the developed model for each technique. The quality of the model was measured using different performance indicators. The goodness of fit between the actual and the predicted stresses values were evaluated using the R value and coefficient of determination (R^2^). The error between the actual and the predicted values of ${\upsigma }_{\mathrm{h}}\mathrm{ and }{\upsigma }_{\mathrm{H}}$ was calculated using the absolute average error (AAPE), root mean squared error (RMSE), variance account for (VAF), and the a^20^ index The correlation coefficient (R) was calculated using Eq. (1), where ${\mathrm{x}}_{\mathrm{i}}$ and ${\mathrm{y}}_{\mathrm{i}}$ are the actual and the predicted ${\upsigma }_{\mathrm{h}}\mathrm{ and }{\upsigma }_{\mathrm{H}}$ gradient values, respectively.

AAPE, RMSE, VAF, a^20^ index and R^2^ were calculated using the following equations, respectively.

2 $$\mathrm{AAPE}=\frac{\sum_{\mathrm{i}=1}^{\mathrm{N}}\mathrm{abs}(\frac{{\mathrm{y}}_{\mathrm{i actual}}-{\mathrm{y}}_{\mathrm{i predicted }}}{{\mathrm{y}}_{\mathrm{i actual}}}\times 100\mathrm{\%})}{\mathrm{N}}$$

3 $$\mathrm{RMSE}=\sqrt{\frac{{\sum_{\mathrm{i}=1}^{\mathrm{N}}({\mathrm{y}}_{\mathrm{i actual}}-{\mathrm{y}}_{\mathrm{i predicted }})}^{2}}{\mathrm{N}}}$$

4 $$\mathrm{VAF}=\left(1-\frac{\mathrm{var}(\left({\mathrm{y}}_{\mathrm{i actual}}-{\mathrm{y}}_{\mathrm{i predicted }}\right) }{\mathrm{var}(\left({\mathrm{y}}_{\mathrm{i actual}}\right)}\right)\times 100$$

5 $${\mathrm{a}}^{20}\mathrm{index}=\frac{{\mathrm{n}}^{20}}{\mathrm{N}}$$

6 $${\mathrm{R}}^{2}=1-\frac{\sum_{\mathrm{i}=1}^{\mathrm{N}}{\left({\mathrm{y}}_{\mathrm{i actual}}-{\mathrm{y}}_{\mathrm{i predicted }}\right)}^{2} }{\sum_{\mathrm{i}=1}^{\mathrm{N}}{\left({\mathrm{y}}_{\mathrm{i actual}}-\frac{\sum_{\mathrm{i}=1}^{\mathrm{N}}{\mathrm{y}}_{\mathrm{i actual}}}{\mathrm{N}}\right)}^{2}}$$

where, ${\mathrm{y}}_{\mathrm{i actual}}$ and ${\mathrm{y}}_{\mathrm{i predicted}}$ are the actual and the predicted output value ( ${\upsigma }_{\mathrm{h}}\mathrm{ and }{\upsigma }_{\mathrm{H}})$ respectively, and N is the total number of data in the dataset. $\mathrm{var}(\left({\mathrm{y}}_{\mathrm{i actual}}-{\mathrm{y}}_{\mathrm{i predicted}}\right)$ is the variance of the difference between the actual and the estimated stress values, while $\mathrm{var}(\left({\mathrm{y}}_{\mathrm{i actual}}\right)$ is the variance of the actual data. In the a^20^ index, the n^20^ is the number of points with the ratio of ${{(\mathrm{y}}_{\mathrm{i actual}}/\mathrm{y}}_{\mathrm{i predicted }})$ is between 0.8 and 1.2. It defines the number of data that estimated the outputs values with a divergence of ± 20% from the actual values.

**Figure 4:**
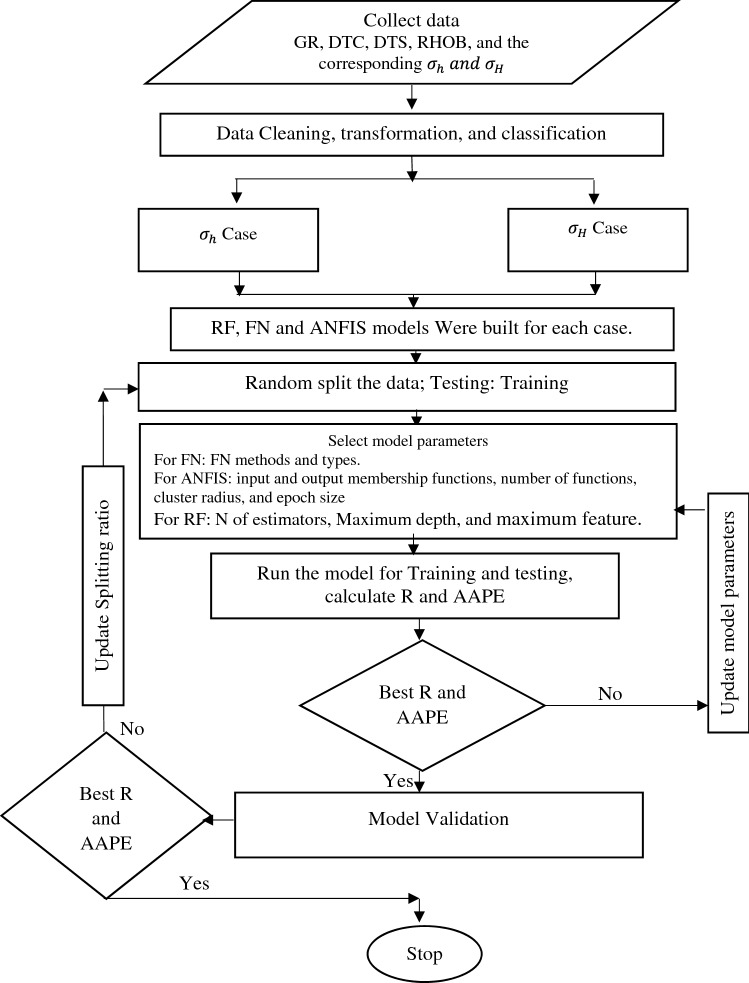
Flowchart for building the different RF, FN, and ANFIS models.

Three different machine learning techniques were applied to the well-log data to predict ${\upsigma }_{\mathrm{h}}\mathrm{ and }{\upsigma }_{\mathrm{H}}$. For each technique, two models were built, one to predict the ${\upsigma }_{\mathrm{h}}$ gradient and the other model to predict the ${\upsigma }_{\mathrm{H}}$ gradient. Figure 4 presents a flowchart for the different model development processes. The data from the first well were randomly split into training and testing datasets. Different splitting ratios were tested varied from 60/40 to 85/15 for the training/testing data points ratio. The model hyperparameters were then optimized to improve the model results. After the model development, the model was verified with unseen data from the dataset from the second well.

RF model comprises many decision trees that accomplish great functioning in a low-dimension dataset. RF overcome the high variance and the overfitting problem by using the single decision tree technique. RF consists of thousands of decision trees, that were built in different bootstrapped data to lower the variance and enhance the power ability of the RF machine. Additionally, during the tree building procedure, a limited number of features are randomly chosen utilizing the cross-validation process, which assists in de-correlate the input trees and consequently improves the model precision^31,32^. Different patterns of RF hyperparameters were applied, including the maximum depth of the tree, the maximum features to be counted when splitting the node in every single tree, and the number of trees that were used to build the forest (N of estimators). The N estimators varied from 3 to 150; the maximum depth had values varied from [3, 4, 5, …, 30]. The maximum feature varied between different three features ["auto", "sqrt", "log2"]. These parameters were optimized to improve the model results and achieve the best indicators (AAPE and R).

The FN technique was provided as an alternative ML tool for ANN. Compared to ANN, FN keeps changing throughout the learning process by adapting to the data^33^. FN does not require weights to be assigned to the neurons because the effect of the weights already exists within the neuron functions from which the outputs obtained are converted to an equivalent output forcefully^33–36^. The neural functions in FN can take various forms chosen from one or more basic families (polynomial and trigonometric, etc.). In the FN, different methods and types can be used to optimize the model parameters for high prediction accuracy. Such methods as functional network forward–backward (FNFBM), functional network backward-forward (FNBFM), and others, while linear and non-linear types can be used in combinations with the FN methods. Similar to RF development, the FN methods and types were optimized to improve the model performance.

ANFIS combines both neural networks and fuzzy logic concepts by depending on a Takagi–Sugeno inference system for data processing applying Boolean logic. After the inputs and outputs are identified, fuzzy if–then rules are used for fitting non-linear regression. ANFIS is optimized through the number of fuzzy rules and epoch size to achieve accurate predictions without overfitting^27–29^. The fuzzy network, cluster radius, and epoch size were optimized for the model performance^37–39^.

## Results and discussion

The three machine learning techniques were used to build six different models, three for ${\upsigma }_{\mathrm{h}}$ prediction and three for ${\upsigma }_{\mathrm{H}}$.

### Minimum horizontal stress prediction

**Figure 5:**
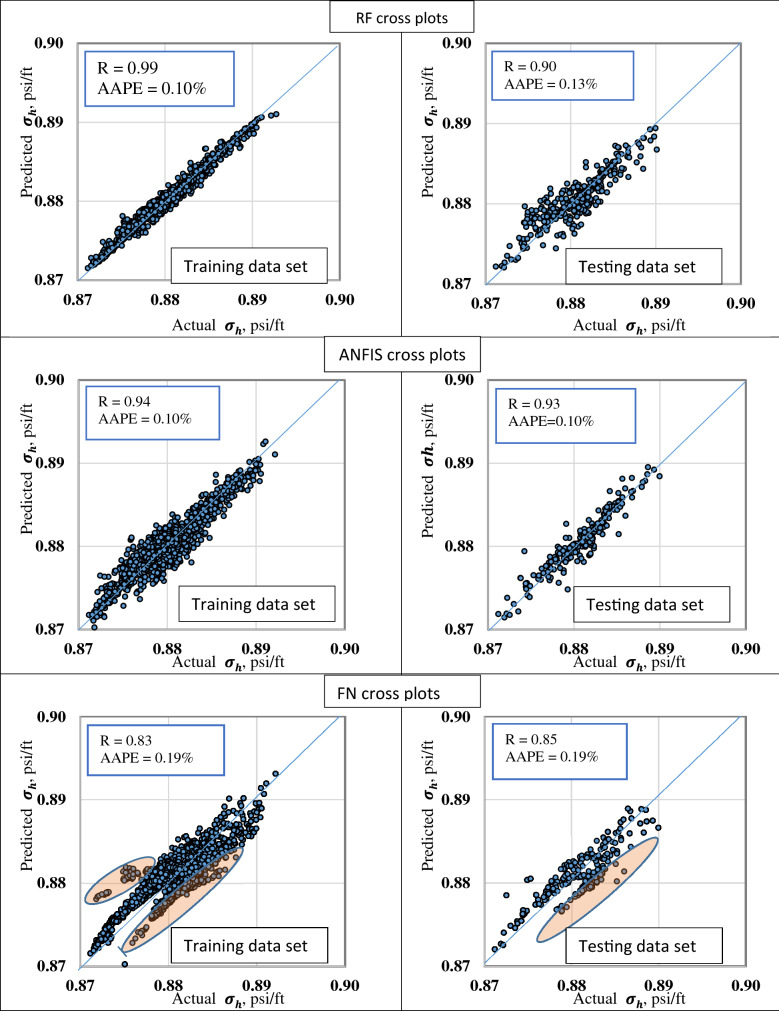
Cross plots of the actual versus the predicted ${\sigma }_{h}$ for the training and the testing datasets from RF, FN, and ANFIS models.

The Well-1 dataset was used to train and test the different ML models. Figure 5 display a cross plot for the actual versus the predicted minimum horizontal stress gradient for RF, ANFIS, and FN.

The RF parameters were optimized to improve the model behavior, as described in Fig. 4. The optimum parameters were N of the estimator is 100, the maximum depth is 25, and the maximum feature is log2. RF cross plot shows that the RF model was capable of estimating accurately ${\upsigma }_{\mathrm{h}}$ from the conventional well-log data. Most of the estimated points were aligned to the 45° line for the training dataset. While the model accuracy marginally reduced for the testing dataset with an R of 0.90. The RF model has R of 0.99 and 0.90 for training, and testing datasets, respectively. AAPE for the RF model was found to be 0.10 and 0.13% for training and testing datasets. GR, DTC, DTS, and RHOB were input to the model. R-value in the case of the testing dataset was found to be 0.88.

To explore the effect of all collected input data on the estimation of the horizontal stresses. Various models with different dataset combinations were developed as shown in Table 2. Excluding NPHI, Ed, and PRd from the input dataset, did not affect the model performance, and GR, DTC, DTS, and RHOB can be used to estimate the horizontal stress.

**Table 2:** Performance of different sets of inputs.

No	Inputs	The validation set using RF Model			
		R	R^2^	AAPE, %	RMSE
1	GR, DTC, DTS, RHOB, NPHI, Ed, PRd	0.9	0.81	0.17	1.9E−3
2	GR, DTC, DTS, RHOB, NPHI, Ed	0.9	0.81	0.15	1.9E−3
3	GR, DTC, DTS, RHOB, NPHI	0.91	0.82	0.13	1.6E−3
4	GR, DTC, DTS, RHOB	0.91	0.83	0.13	1.6E−3
5	GR, DTS, RHOB	0.91	0.82	0.13	1.6E−3

Similarly, the ANFIS technique was applied to the dataset from Well-1 to train and test the model. ANFIS parameters were optimized. The optimal input and the output membership function are gaussmf and linear, respectively. The optimal cluster radius and epoch size are 0.1 and 10, respectively. The ANFIS cross plot in both training and testing datasets shows the capability of the ANFIS model to predict ${\upsigma }_{\mathrm{h}}$ from the conventional well-log data with a good alignment with the 45° line. ANFIS model slightly outperforms RF model with less overfitting effect. Both training and testing datasets have AAPE of 0.10 with R values of 0.94 and 0.93 in the cases of training and testing datasets.

The FN was then implemented on the dataset from Well-1. Different combinations of FN methods and types were used to optimize the developed model performance. Table 3 summarizes FN methods and types and the corresponding R values and AAPE from the testing dataset. The different combinations showed that the FN model was less performed compared to RF and ANFIS models with optimum R values of 0.83 and 0.85 for the training and testing datasets, respectively. Most of the data aligned to the 45° line, with some points deviated from it. AAPE was estimated to be 0.19% in both training and testing datasets.

**Table 3:** Different FN methods and types to optimize the developed model performance with AAPE and R-values for the testing dataset.

Method	'FNBFM'	'FNFBM'	'FNBEM'	'FNFBM'
Type	'Non-Linear(2)'	'Non-Linear(2)'	'Non-Linear(2)'	'Non-Linear(1)'
AAPE %	0.19	0.193	0.23	0.24
R	0.85	0.85	0.79	0.78

**Figure 6:**
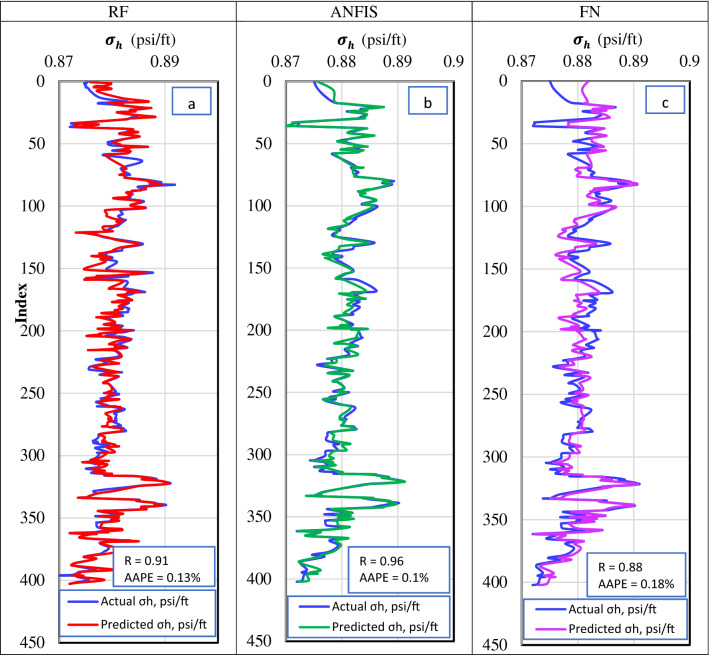
Actual versus the predicted ${\sigma }_{h}$ for the validation dataset from (**a**) RF model, (**b**) ANFIS model, and (**c**) FN model.

The dataset from Well-2 was used to validate the different ML models for ${\upsigma }_{\mathrm{h}}$ prediction. Figure 6 shows the actual versus the predicted ${\upsigma }_{\mathrm{h}}$ gradient for the validation dataset. In both RF and ANFIS models, the graph shows that the actual and estimated values are almost identical, which proves the ability of RF and ANFIS models to predict accurately ${\upsigma }_{\mathrm{h}}$ gradient from well-log data. FN model was capable of describing the general trend of the ${\upsigma }_{\mathrm{h}}$ gradient with marginal deviation from the actual values at some locations, particularly at the spikes.

Table 4 summarizes the quality indicator for RF, ANFIS, and FN models for the different datasets. It shows that the ANFIS model has the best performance with R and AAPE values are almost the same for training, testing, and validation datasets. RF displays overfitting behavior slightly where the training R value was 0.99 that decreased to 0.90 with the testing dataset and 0.91 in the validation dataset. FN had the lowest accuracy with an R factor of 0.85 and 0.88 for the testing and validation datasets. The calculated a^20^ index for the three ML techniques showed 100%, which indicates that all the predicted stresses were with 20% deviation from the actual ${\upsigma }_{\mathrm{h}}$ gradient.

**Table 4:** Performance indicators summary for the training, testing, and validation datasets from the RF, ANFIS, and FN models to predict ${\sigma }_{h}$ from well-logs.

	RF			ANFIS			FN		
	Train	Test	Val	Train	Test	Val	Train	Test	Val
R	0.99	0.90	0.91	0.94	0.93	0.96	0.83	0.85	0.88
R^2^	0.97	0.82	0.83	0.88	0.86	0.86	0.7	0.72	0.77
AAPE%	0.10	0.13	0.13	0.10	0.10	0.10	0.19	0.19	0.18
RMSE	6E−4	1.5E−3	1.6E−3	1.3E−3	1.2E−3	1.4E−3	2E−3	2E−3	1.5E−3
VAF%	97	81.6	82	88	86.6	86.1	69.1	72	77.4
A20%	100	100	100	100	100	100	100	100	100

### Maximum horizontal stress prediction

**Figure 7:**
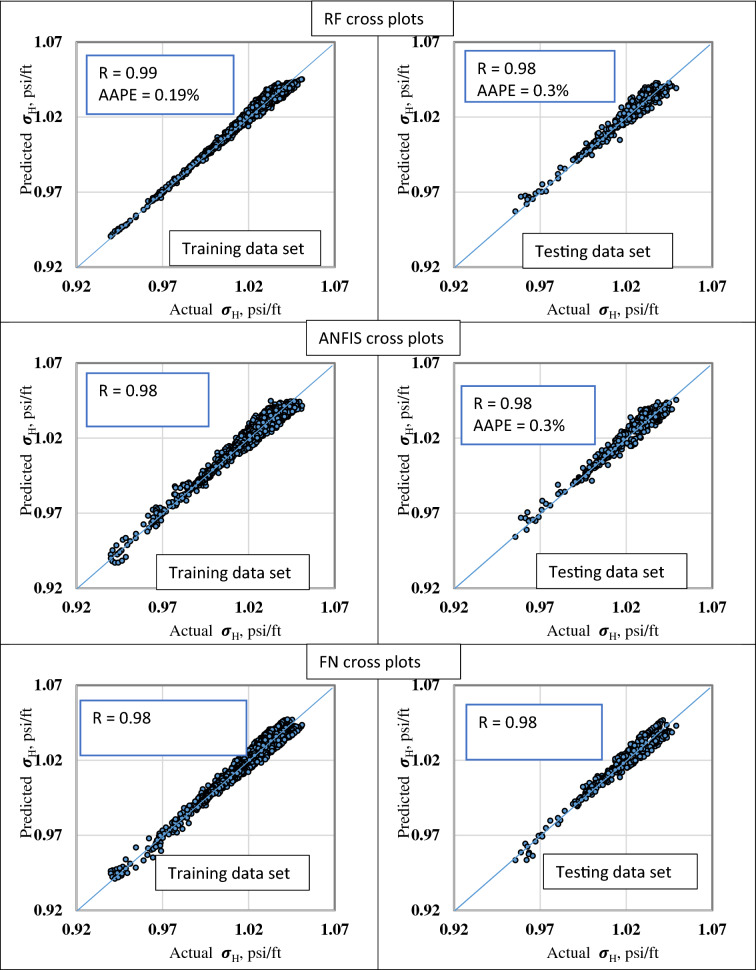
Cross plots of the actual versus the predicted ${\sigma }_{H}$ for the training and the testing datasets from RF, FN, and ANFIS models.

The Well-1 logging data, including GR, DTC, DTS, and RHOB as inputs and the ${\upsigma }_{\mathrm{H}}$ gradient as output were used to train and test the RF, ANFIS, and FN models. Figure 7 presents the actual versus the estimated ${\upsigma }_{\mathrm{H}}$ cross plots for the training and testing datasets for the RF, ANFIS, and FN models.

The RF parameters were optimized to improve the model performance. The optimum N of the estimator, maximum depth, and maximum feature were 100, 20, and log2. Similarly, the optimal parameters for the ANFIS input and the output membership functions were found to be gaussmf and linear, whereas the cluster radius was 0.1, and the epoch size was 15. The best combination of the FN method and type was found to be the FNBFM and non-linear (2).

Figure 7 shows plots of the actual versus the estimated ${\upsigma }_{\mathrm{H}}$ gradient of the RF, FN, and ANFIS models for both training and testing datasets. The three model presents a very accurate prediction of ${\upsigma }_{\mathrm{H}}$ from the conventional well logs with all the data lined up with the 45° line. The R values for the three models' training and testing dataset were higher than 0.98, with AAPE less than 0.3%.

**Figure 8:**
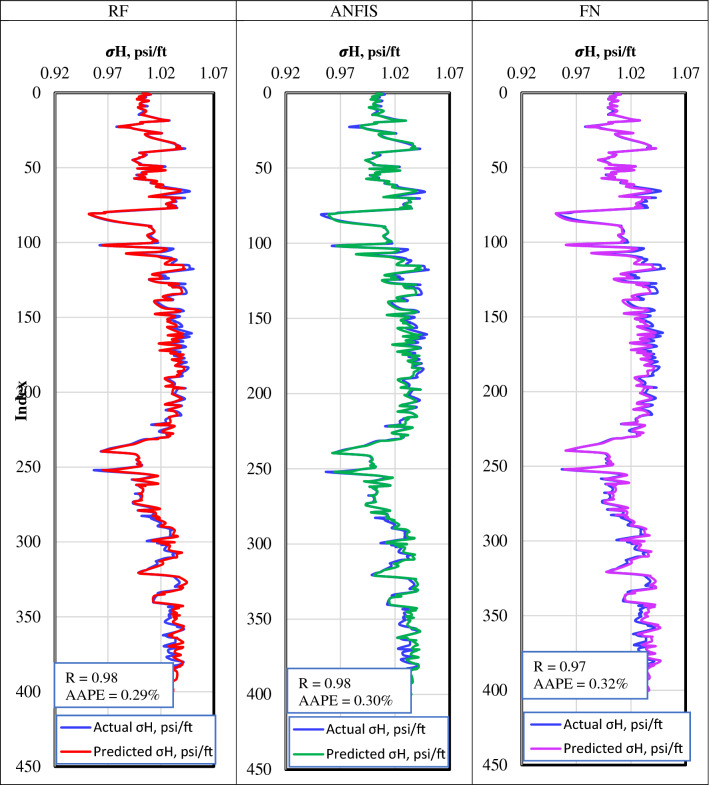
Actual versus the predicted ${\upsigma }_{\mathrm{h}}$ gradient for the validation dataset from (**a**) RF model, (**b**) ANFIS model, and (**c**) FN model.

The Well-2 dataset was used to validate the RF, ANFIS, and FN developed models that predict ${\upsigma }_{\mathrm{H}}$ gradient. Figure 8 shows the actual versus the predicted ${\upsigma }_{\mathrm{H}}$ gradient for the validation dataset. In the RF, ANFIS, and FN models, the actual and predicted ${\upsigma }_{\mathrm{H}}$ gradient curves are identical, which confirms the three models' prediction accuracy.

Table 5 summarizes the quality indicator for RF, ANFIS, and FN models for the training, testing, and validation datasets. It shows the three models' optimal robust performance where the R and AAPE values are almost the same for the different datasets. R-value for the different models was higher than 0.97 in all datasets with AAPE of 0.3% and RMSE of 3.9E-6. The a^20^ index was found to be 100% for the different datasets with the different models.

**Table 5:** Performance indicators summary for the training, testing, and validation datasets from the RF, ANFIS, and FN models to predict ${\sigma }_{H}$ from well-logs.

	RF			ANFIS			FN		
	Train	Test	Val	Train	Test	Val	Train	Test	Val
R	0.99	0.98	0.98	0.98	0.98	0.98	0.98	0.98	0.97
R^2^	0.98	0.97	0.96	0.94	0.98	0.96	0.92	0.98	0.95
AAPE%	0.19	0.3	0.29	0.3	0.3	0.30	0.3	0.3	0.32
RMSE	2.6E−3	3.2E−3	3.6E−3	3.7E−3	3.8E−3	3.8E−3	4.2E−3	3.9E−3	3.9E−3
VAF%	98	97.3	95.7	93.8	98.4	95.6	91.9	98.2	95.4
A20%	100	100	100	100	100	100	100	100	100

The established ML models were able to estimate the ${\upsigma }_{\mathrm{h}}$ and ${\upsigma }_{\mathrm{H}}$ gradients from the logging data; GR, DTC, DTS, and RHOB. Hence, a continuous profile for the ${\upsigma }_{\mathrm{h}}$ and ${\upsigma }_{\mathrm{H}}$ with depth can be obtained. Having continuous profiles of the least principal stresses for the drilled wells provides practical solutions to several wellbore instability issues, which assist well integrity and better hydraulic fracturing design.

It should be emphasized that the application of the developed models is limited to carbonate formations because other formations may have different log responses to the geomechanical properties that control the horizontal stresses values. Moreover, similar to any developed model, it should be applied to input data within the model's inputs ranges in Table 1 to guarantee trustworthy results. For future work, a similar analysis will be conducted on a dataset for a sandstone formation to evaluate the application of ML techniques to predict the ${\upsigma }_{\mathrm{h}}$ and ${\upsigma }_{\mathrm{H}}$ in sandstone formations.

## Conclusions

This study presents the application of RF, ANFIS, FN techniques to predict the minimum and maximum horizontal stress gradients from the convention well-logs, including GR, DTC, DTS, and RHOB. Following are the main conclusions;

- The GR, DTC, DTS, and RHOB logs showed a strong correlation with ${\upsigma }_{\mathrm{h}}$ and ${\upsigma }_{\mathrm{H}}$.
- RF and ANFIS models were able to predict accurately ${\upsigma }_{\mathrm{h}}$ and ${\upsigma }_{\mathrm{H}}$ gradients from the conventional well-logs with AAPE less than 0.3%.
- FN model accurately predicted ${\upsigma }_{\mathrm{H}}$ gradient from the well-logs but did not provide the same accuracy in predicting ${\upsigma }_{\mathrm{h}}$ gradient.

This study showed the capabilities and robustness of machine learning techniques to predict the minimum and maximum horizontal stress gradients from the conventional well-logs with about ± 0.3% accuracy error and goodness of fit (R) of 0.90–0.98.

## List of symbols

AAPE: Average absolute percentage error
ANFIS: Adaptive neuro-fuzzy inference system
ANN: Artificial neural network
DFIT: Diagnostic fracture injection test
DTC: Compressional waves transit time, µs/ft
DTS: Shear waves transit time, µs/ft
FN: Functional network
FNFSM: Functional network forward-selection method
FNESM: Functional network exhaustive-search method
FNBEM: Functional network backward-elimination method
FNFBM: Functional network forward–backward method
FNBFM: Functional network backward-forward method
GR: Gamma-ray, API
LOT: Leak-off test
N: The number of data points
ML: Machine learning
R: Correlation coefficient
RF: Random forest
RHOB: Formation bulk density, g/cm^3^
${\mathrm{x}}_{\mathrm{i}}$: The independent parameter
XLOT: Extended leak-off test
${\mathrm{y}}_{\mathrm{i}}$: The dependent parameters
${\upsigma }_{\mathrm{V}}$: Overburden stress, psi/ft
${\upsigma }_{\mathrm{h}}$: Minimum horizontal stress, psi/ft
${\upsigma }_{\mathrm{H}}$: Maximum horizontal stress, psi/ft
${\upsigma }_{\mathrm{x}}\mathrm{ and }{\upsigma }_{\mathrm{y}}$: The standard deviation for the independent and dependent parameters
${\upmu }_{\mathrm{x}}\mathrm{ and }{\upmu }_{\mathrm{y}}$: Mean for the independent and dependent parameters
